# Distributions of soil branched glycerol dialkyl glycerol tetraethers from different climate regions of China

**DOI:** 10.1038/s41598-019-39147-9

**Published:** 2019-02-26

**Authors:** M. Wang, Z. Zheng, Y. Zong, M. Man, L. Tian

**Affiliations:** 1 Guangdong Provincial Key Laboratory of Geodynamics and Geohazards, School of Earth Sciences and Engineering, Sun Yat-sen University, Guangzhou, China; 2 Department of Earth Sciences, The University of Hong Kong, Hong Kong, SAR China

## Abstract

Distributions of branched glycerol dialkyl glycerol tetraethers (brGDGTs) from soil bacteria have been recently used for reconstructions of past soil pH and air temperature history. Here, we report the brGDGTs distribution patterns from different climate regions of China, using 42 novel brGDGT data from sub-tropical Chinese soils, compiled alongside previously-published data encompassing different environmental conditions across China. These patterns show high abundance of Ia fraction in soils from humid areas, and high abundance of IIa′ and IIIa′ fractions corresponds to semi-humid or semi-arid conditions, implying a possible relationship with precipitation. The abundance of IIa fraction appears negatively correlated with air temperature. Statistically, the soil pH and temperature factors represent 62% and 30% variations of the total variance respectively. For soils from humid and semi-humid regions, strong correlations between cyclization of branched tetraethers (CBT′) index and soil pH (R^2^ = 0.89) and between methylation of branched tetraethers (MBT′_5ME_) index and mean annual temperature (MAT)(R^2^ = 0.82) exist. When soils from semi-arid regions are included, the correlations become slightly stronger with pH and significantly weaker with MAT. Our study confirms the usefulness of soil brGDGTs distributions for Quaternary palaeo-climate reconstructions.

## Introduction

Understanding of the modern relationship between climatic parameters and environmental proxies is key to the reconstructions of past climate and environmental histories. Thus, finding suitable environmental proxies has been an important task for the past decades. For continental environments, various proxies such as pollen^1,2^, diatoms^3,4^, ostracods^5,6^, chironomids^7^, fossil plant assemblages^8^ and stable isotope (e.g. δ^13^C and δD)^9–11^ have been examined and applied to Quaternary palaeo-environmental reconstructions. However, most of the existing proxies contain large uncertainty, and/or they are not quantitatively related to air temperature and precipitation, the two climatic variables most commonly used to defining climate zones^12^. Hence, a search for suitable proxies for these two climatic variables has continued.

Recently, using the cyclisation ratio of branched tetraethers (CBT) and methylation index of branched tetraethers (MBT) from branched Glycerol dialkyl glycerol tetraethers (brGDGTs) to reconstruct past soil pH (as a proxy for precipitation) and air temperature has been attempted. This is because the CBT and MBT indices correlate well with soil pH and air temperature respectively^13–22^. The brGDGTs are membrane lipids that are ubiquitous in lacustrine, estuarine and marine environments^13,14^. Accordingly, the CBT and MBT indices from soils^15,16^, peat^17–19^ and sediments^20–22^ have been examined. About 10 years ago, Weijers^15^ analyzed 134 globally distributed soils from more than 90 regions and developed calibrations between MBT-CBT and mean annual temperature (MAT) and between the CBT and soil pH. Subsequently, Peterse^23^ revised the calibrations by extending the dataset to 278 globally distributed soils.

In addition, De Jonge^24^ improved the liquid chromatography method and proposed MBT′_5ME_ and CBT′ indices by separating 5-methyl and 6-methyl brGDGTs. The 6-methyl brGDGTs are denoted by a prime after the roman numerals for their corresponding 5-methyl isomers. The newly developed proxies^24–26^ indicate that MBT′_5ME_ is correlated well with MAT, whilst CBT′ has a strong relationship with soil pH. Following the separation of 5- and 6- methyl brGDGTs, an improved correlation was achieved, as well as the increased accuracy^19^ in comparison to the old calibrations^23^. Using this new method, Naafs^17^ developed peat-specific MBT′_5ME_-MAT and CBT′_peat_-pH calibrations. Furthermore Wang^27^ and Naafs^28^ produced MBT′_5ME_ soil temperature calibrations for North China and the world respectively. However, studies of separated 5-methyl and 6-methyl brGDGTs analysis are insufficient in subtropical China in comparison to those from northern and northwestern China^27,29–34^.

**Figure 1:**
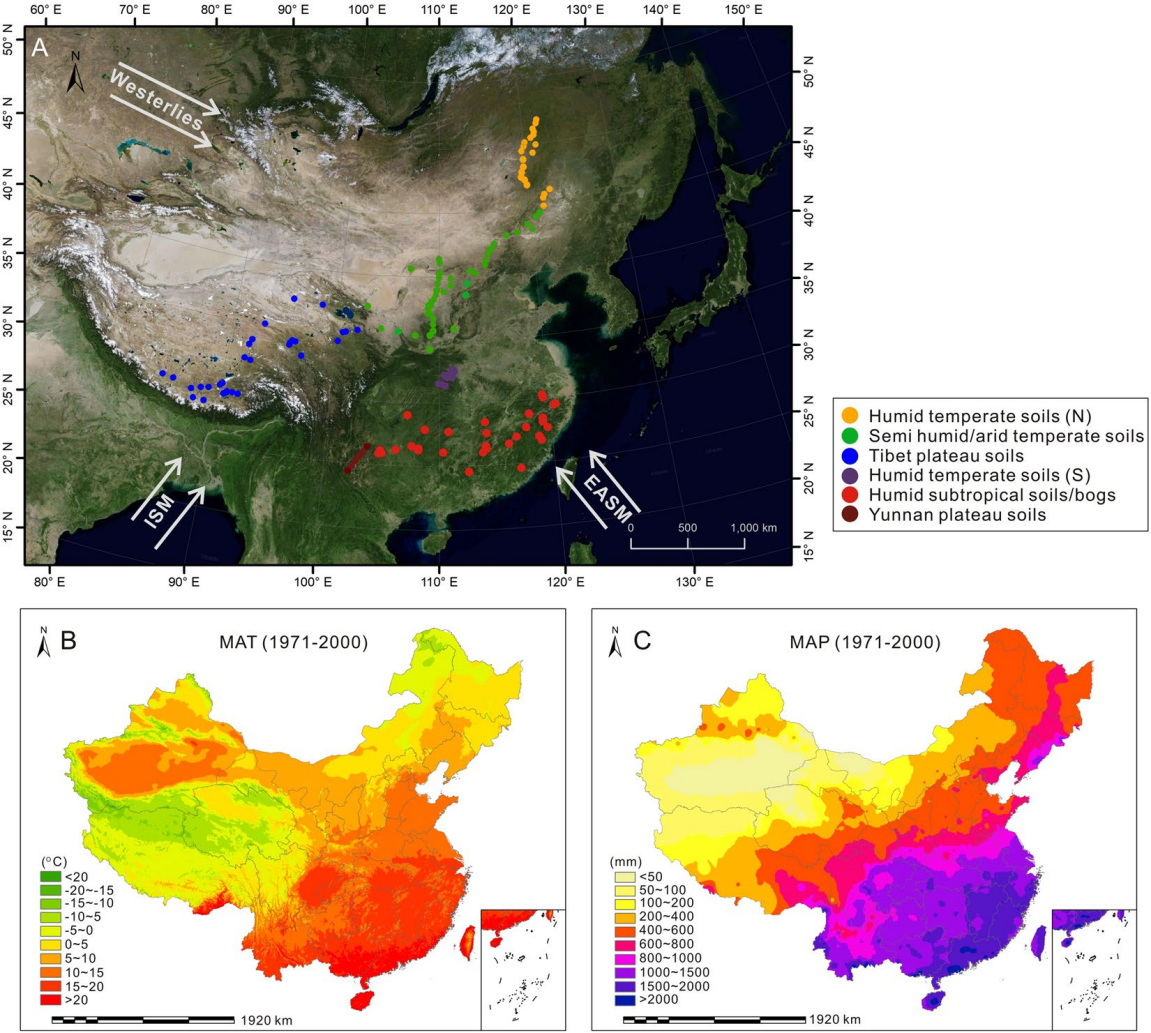
Sampling locations and climate conditions across China. Map A shows locations of 58 soil samples (orange) collected by Wang^27^ and Xiao^29^ from northeast China (group IV), 102 soil samples (green) collected by Wang^27^ and Xiao^29^ from northern China (group V), 42 soil samples (blue) collected by Ding^30^ and Xiao^29^ from Qinghai-Tibetan Plateau of China (group IV), 52 soil samples (purple) collected by Yang^32^ and lei^31^ from central China (group III), 13 soil samples (dark red) collected by Lei^31^ from Yunnan Plateau, southwest China (group II), and 47 soil and bog samples (bright red) collected by this study from southern China (group I). Map B illustrates spatial variations of mean annual temperature (C°) measured between 1971 and 2000. (**C**) Map C denotes the spatial variations of mean annual precipitation (mm) measured between 1971 and 2000. The MAP A was produced using ArcGIS, version number 10.5, http://esrichina.hk/ (The HKU has purchased the copyright of ArcGIS 10.5. The satellite imagery in map A were obtained from Google Earth, version number 7.3.1.4507, https://www.google.com/earth/download/gep/agree.html). Map data: Google, DigitalGlobe. The MAT and MAP data sets are provided by the Data Center for Resources and Environmental Sciences, Chinese Academy of Sciences (http://www.resdc.cn MAT: http://www.resdc.cn/data.aspx?DATAID=154 MAP: http://www.resdc.cn/data.aspx?DATAID=153).

This separated 5-methyl and 6-methyl brGDGTs^24–26^ can provide further details of brGDGTs distributional patterns. With these details, the brGDGTs relationship with soil pH and air temperature can be further evaluated. In this paper we examine soil brGDGTs distributional patterns from a wide area of East Asian continent that encompasses humid subtropical, warm and cold temperate, semi-arid and high plateau environments (Fig. 1). Such diverse environments allow us to examine the variability of brGDGTs patterns between climate zones. Over the past few years, certain amount of brGDGT data from surface soils across this region has been generated^27,29–34^. In this paper we add to the data set our new data from subtropical China. A total of 293 soil brGDGT data (Fig. 1) presented here provides a clearer understanding of the brGDGT distribution patterns in global peat and mineral soils, and aids the use and interpretation of CBT and MBT indices in the palaeo-record.

### Effects of separated 5-methyl and 6-methyl brGDGTs on subtropical soils

**Figure 2:**
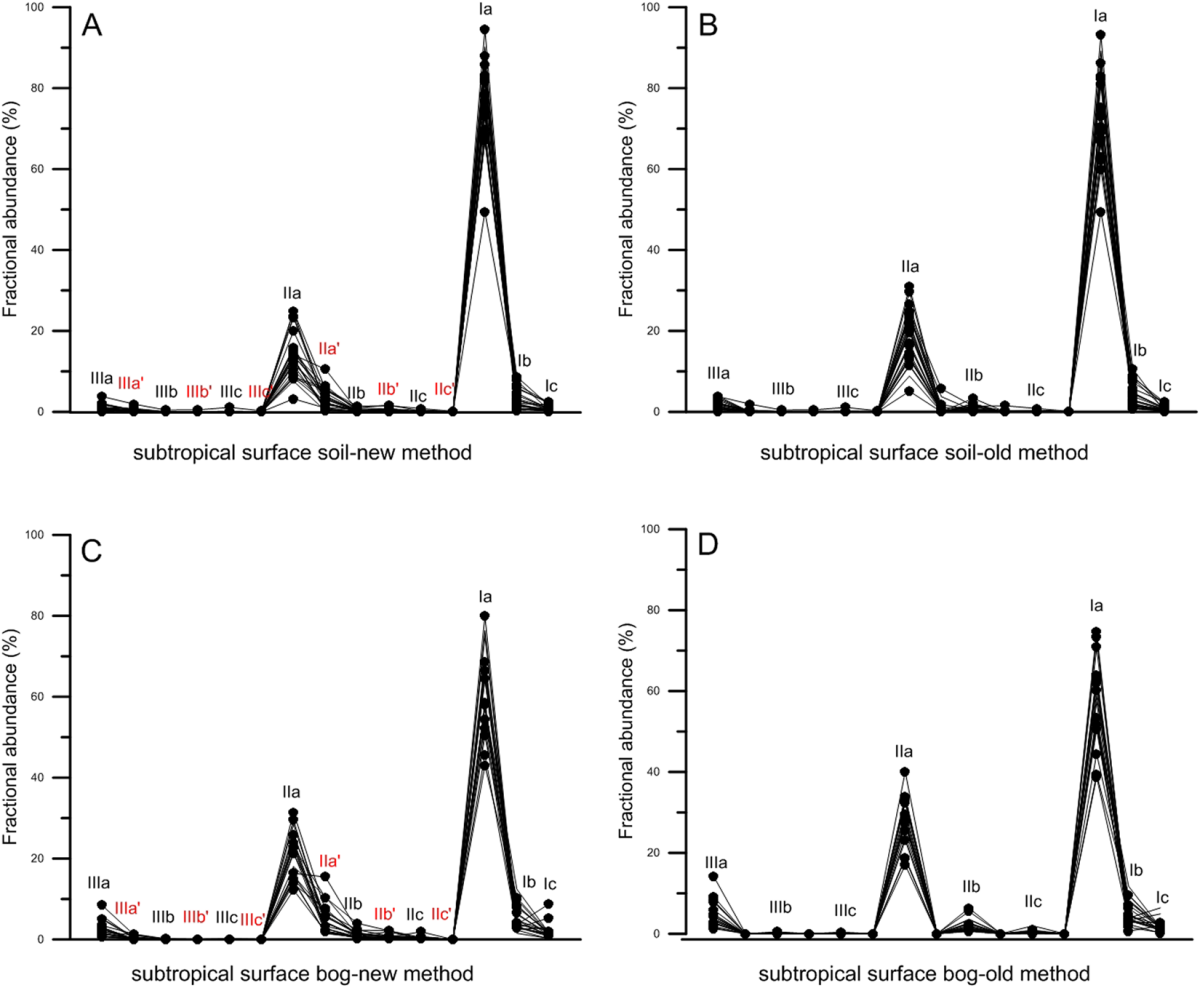
Comparisons of brGDGT distribution between soil samples and bog samples collected by this study and analyzed with the new method and the old method. (**A**) Subtropical soil samples analyzed with the new method. (**B**) Subtropical soil samples analyzed with the old method. (**C**) Subtropical bog samples analyzed with the new method. (**D**) Subtropical bog samples analyzed with the old method. (**A**,**C**) show the abundance of 15 brGDGT fractions. (**B**,**D**) reveal the abundance of 9 brGDGT fractions. Fractional abundance is expressed as percentage of the total brGDGTs.

In order to reveal the potential improvement of the separated 5-methyl and 6-methyl brGDGTs on Chinese subtropical sediments, we have analyzed all the subtropical samples with a LC-MS equipped with one-cyano-column^23^ and the two-silica-column^24–26^ methods. The result (Fig. 2) shows that separated 5-methyl and 6-methyl brGDGTs can provide further details of the brGDGT-IIIa′, brGDGT-IIIb′, brGDGT-IIIc′, brGDGT-IIa′, brGDGT-IIb′ and brGDGT-IIc′ fractions, which has been reported by De Jonge *et al*.^24^, Hopmans *et al*.^25^ and Freymond *et al*.^26^. There is not significant difference in the brGDGTs distribution patterns between the two methods. Overall the results of both methods indicate that the most abundant brGDGTs are the Ia and IIa fractions, with some low abundant brGDGT-Ib, brGDGT-IIb and brGDGT-IIIa fractions. However, the separated 5-methyl and 6-methyl brGDGTs^24–26^ reveals abundance of the brGDGT-IIa′ fraction (0.3% to 15.5%), which provides more brGDGT distribution details when compared with data from other climate zones.

**Figure 3:**
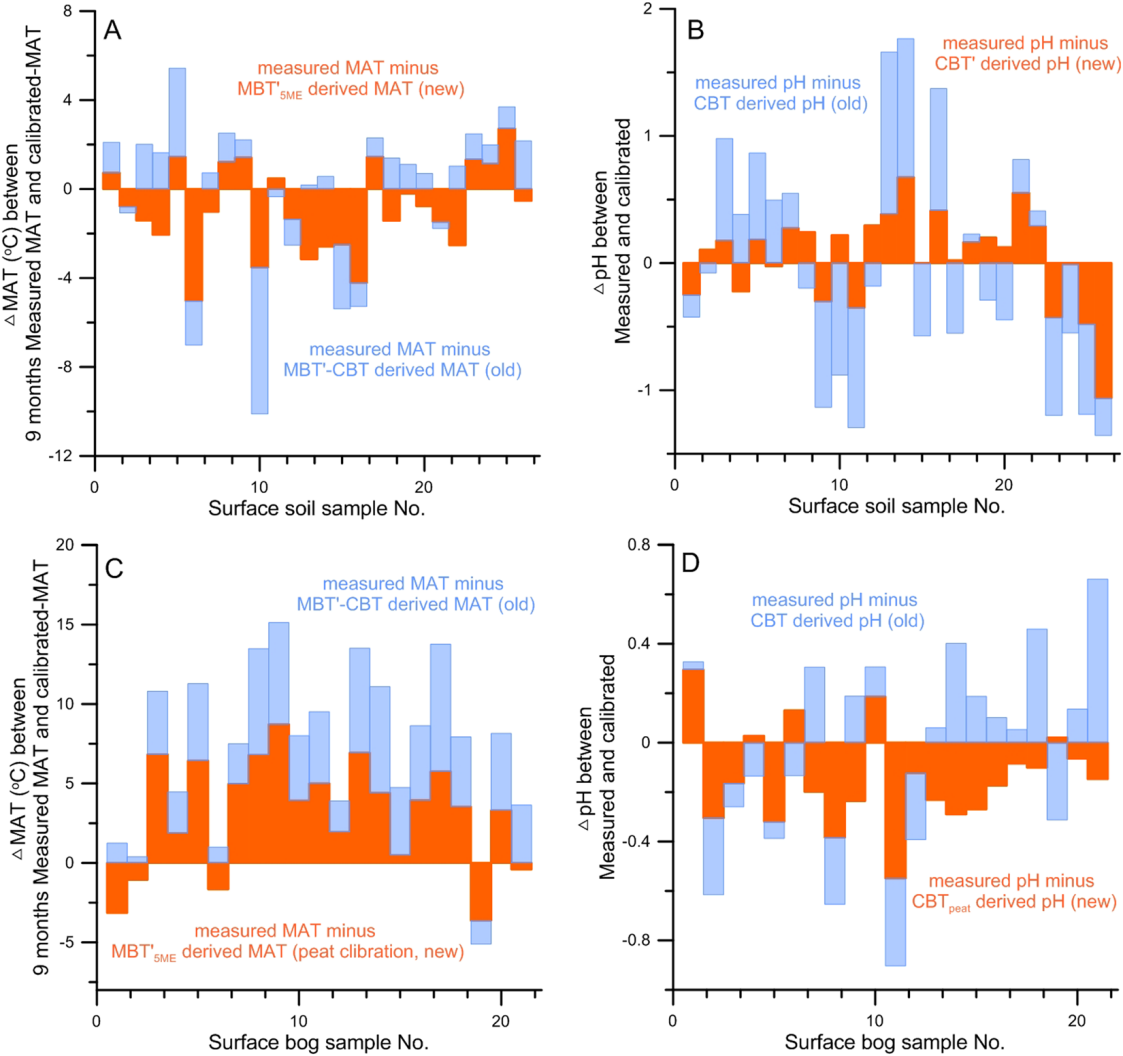
Comparisons of the calibrated MAT (C°) and pH analyzed with the new (orange bars) and old (blue bars) methods for the soil and bog samples. (**A**) Deviations of the soil brGDGTs calibrated MAT (C°) from the observed 9-month MAT (C°). (**B**) Deviations of the soil brGDGTs calibrated pH from the observed pH. (**C**) Deviations of the bog brGDGTs calibrated MAT (C°) from the observed 9-month MAT (C°). (**D**) Deviations of the bog calibrated pH from the observed pH.

Differences in brGDGT distribution exist between the soil and bog samples collected from humid subtropical China are apparent in both methods. This is in accordance with the suggestion by Naafs^17^ that peat-specific brGDGTs calibrations are different in peats relative to soils. Finally, Fig. 3 shows the results of a further examination that helps compare the precision of both methods when they are applied to the pH and MAT reconstructions. As seen from the plots, the two-silica-column method produces reconstructions with a significantly smaller range of uncertainty than the one-silica-column method. This applies to both soil and bog samples.

### Distribution patterns of brGDGTs between climate zones

**Figure 4:**
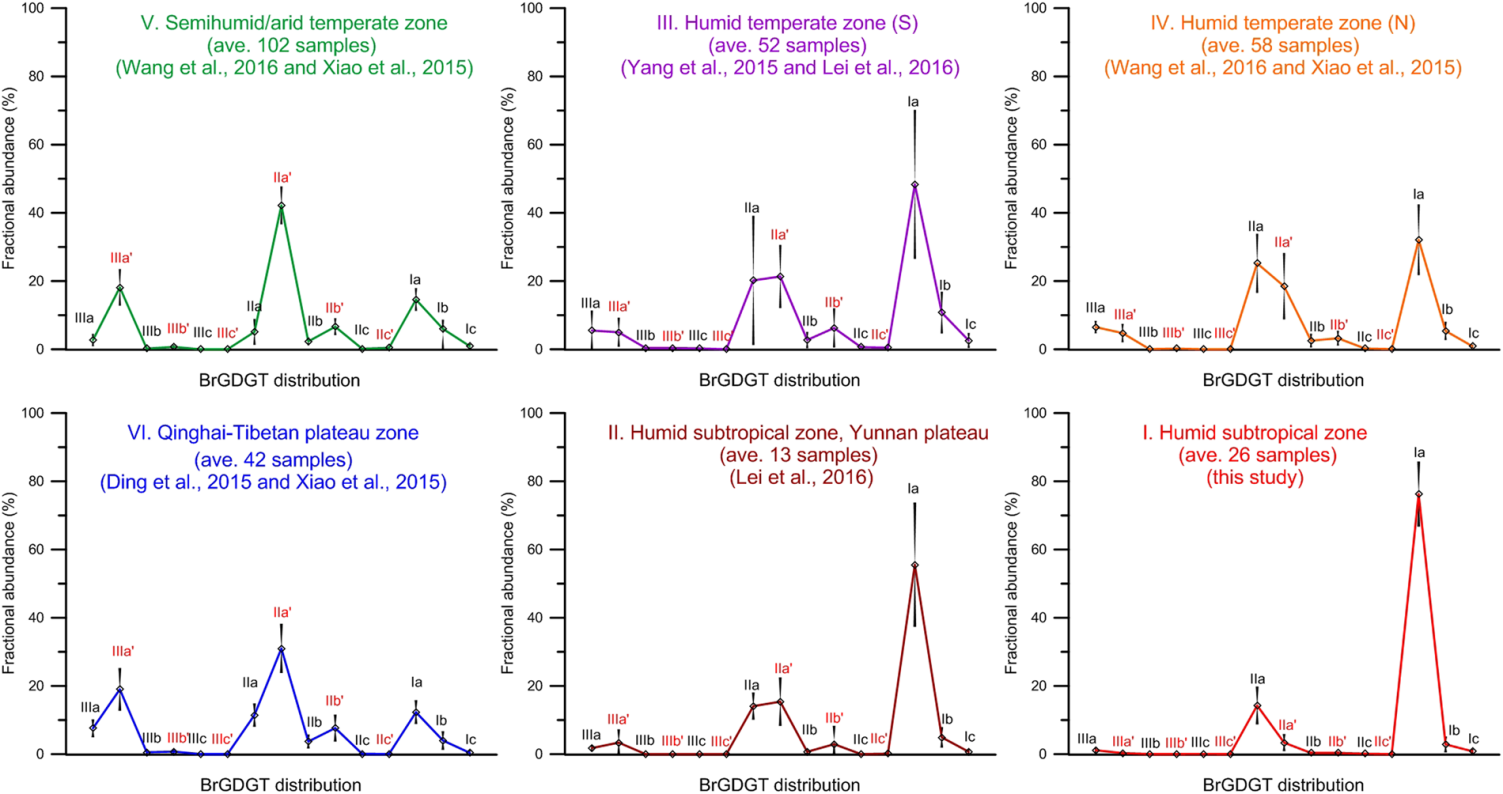
The brGDGTs distributions from different climate regions across China. Samples are divided into six groups (from I to VI) according to their location and climate conditions (Fig. 1). Each plot presents the average abundance of each brGDGT fraction from the same group. Standard deviations of brGDGTs abundance are indicated for each fraction.

Here, all the surface soil samples are combined and analyzed for their brGDGTs distribution patterns. Based on their similarity and geographical locations, the 293 samples (Fig. 1A) are divided into six groups (Fig. 4) in order to investigate their correlations with climate conditions^1^. According to the relative abundance of each fraction in the brGDGTs distribution patterns, the 6 groups are further summarized in 2 clusters for discussion.

Cluster A includes groups I, II, III and IV, which are from the humid to semi-humid half of China (Fig. 1A). Group I is represented by 26 soil samples collected by this study from the humid subtropical southern China. This region is under a warm (MAT over 18 °C) and humid (mean annual precipitation, MAP over 1600 mm) climate (Fig. 1B,C). Within this region, the soil brGDGTs are dominated by brGDGTs-Ia (76%), followed by IIa fraction (14%). The Ib, IIa′ and IIIa fractions appear in very low abundance (1%~3%), whilst all the remaining fractions are close to zero. Nevertheless, this group is characterized by the high abundance of Ia and IIa fractions.

Group II is from subtropical southwest China, the Yunnan Plateau with an average altitude over 1000 m above sea level^31^. In the sampling area the local MAP and MAT are around 800 mm and 14–18 °C respectively. Group III is from a small region of southern temperate China (Fig. 1A), where the local MAP varies between 800 mm and 1000 mm and the local MAT is around 16–18 °C. In other words, the MAP of these two sampling areas is significantly lower than that of Group I^35^. Comparatively, the Ia abundance of groups II (56%) and III (48%) is slightly lower than that of Group I (76%) (Fig. 4). On the other hand, the IIa abundance of groups II (14%) and III (20%) is slightly higher than that of Group I (14%). The most important difference between these three groups appears in the IIa′, IIIa and IIIa′ fractions. These fractions are exceptionally low in Group I (Fig. 4).

Group IV is located in the northern temperate China^27,29^. The MAP there is much lower, around 400–600 mm, and the MAT is also low, only around 2 °C, both are significantly lower than groups I, II and III. Comparatively, the Ia abundance of Group IV is significantly lower (32%). On the other hand, the abundance of IIa fraction in this group is the highest (25%) within Cluster A. The IIa′, IIIa and IIIa′ fractions in Group IV are also important.

Cluster B comprises Groups V and VI (Fig. 4). Group V includes sampling sites on the inland side of the northeast-southwest trending mountain ranges^27,29^ (Fig. 1A) along the so-called Asian summer monsoon margin^36^. Along this margin, the MAP drops significantly to below 600 mm, and the MAT is also reduced to below 12 °C (Fig. 1B,C). Furthermore, these sampling sites are under the influence of the Westerlies^37^, and as a result, strong evaporation occurs in these sites^38^. Soil samples of Group VI are collected from the Qinghai-Tibetan Plateau^29,30^, where both of the MAP and MAT are very low, i.e. below 600 mm and below 10 °C respectively (Fig. 1). These sites are also strongly influenced by the Westerlies^37^. The abundance of Ia fraction in Groups V (15%) and VI (12%) is significantly lower than the other four groups (Fig. 4). The abundance of IIa fraction is also very low (5% for Group V and 11% for Group VI). In contrast, the IIa′ and IIIa′ fractions appear highly abundant in the brGDGTs distributions of groups V (IIa′ 42% and IIIa′ 18%) and VI (IIa′ 30% and IIIa′ 18%).

The variations in soils brGDGT distributions under different environmental conditions may represent a physiological adaptation of soil (acido-) bacteria in response to changes in external environmental conditions. Alternatively, the difference in soil brGDGT distributions may suggest a change in the fundamental community of soil bacteria. Although beyond the scope of this data, in order to better constrain this, further dual lipid-DNA based studies are required. Nevertheless, for the scientific community working on paleo-climate reconstructions, the above results confirm the usefulness of soil brGDGTs for paleo-climate reconstructions in humid to semi-arid regions, because the relative abundance of several brGDGT fractions correlates well with environmental conditions such as MAT and MAP.

### Controlling factors, the CBT′ and MBT′_5ME_ indices

As discussed above, the different brGDGTs distribution patterns correspond to different climate conditions. It is therefore logical to assume that a statistical relationship between climate parameters (MAP and MAT) and the brGDGTs exists. To test the strength of this relationship, a factor analysis is performed using the most abundant brGDGTs fractions (Ia, IIa, IIa′ and IIIa′ that contribute 72.0% of total variations), the two most commonly used climate parameters (MAP and MAT), soil pH and the two brGDGT indices (CBT′ as the pH proxy and MBT′_5ME_ as the air temperature proxy). The result shows that the Kaiser-Meyer-Olkin (KMO) value of the factor analysis is 0.84, indicating a high correlation between the variables used. The Bartlett-test of sphericity (*p*) is lower than 0.01, suggesting that all the variables are suitable for the factor analysis.

**Figure 5:**
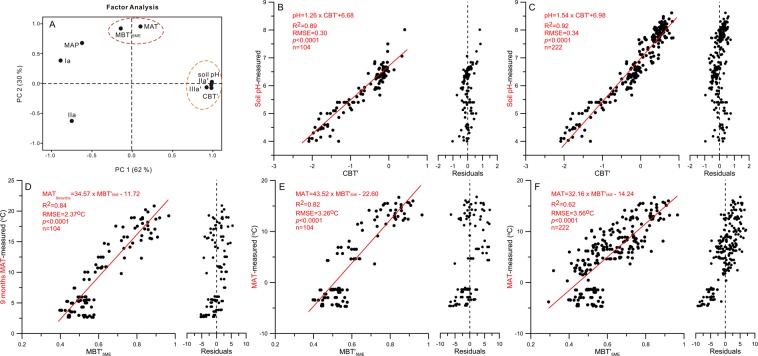
Factor analysis and regressions. Graph A shows the result of the factor analysis for all the soil samples collected across China. Variables used are MAP, MAT, soil pH, CBT′, MBT′_5ME_ and abundance of brGDGT fractions (Ia, IIa, IIa′ and IIIa′). Graph B reveals the regression between the calibrated CBT′ index and the observed soil pH values of the samples from humid to semi-humid regions of China (groups I, II, III and IV, Fig. 1). Graph C explains the relationship between the calibrated CBT′ index and the observed soil pH values from soil samples of all six groups. Graphs D and E illustrate the regressions between the calibrated MBT′_5ME_ and the observed MAT and MAT_9months_ of the soil samples from humid to semi-humid regions of China (groups I, II, III and IV, Fig. 1). Graph F reports the correlation between the calibrated MBT′_5ME_ and the observed MAT based on samples from all six groups. MAT_9months_ is the average MAT from March to November.

The result of the factor analysis (Fig. 5A) shows that the first Principal Component (PC 1) contributes 62% to the total variations and corresponds to relative abundance of Ia fraction in contrast to IIa′ and IIIa′ fractions. PC1 aligns clearly with CBT′ index and soil pH, and confirms a strong relationship between them. Specifically, high abundance in IIa′ and IIIa′ fractions is related to high pH or more alkaline conditions. The correlation with soil pH is very strong (Fig. 5B,C) as indicated by the high correlation coefficient (R^2^ = 0.89) and the small random mean square of errors (RMSE = 0.30) for the 104 soil samples from the humid to semi-humid regions. If the soil samples from the two semi-arid regions are included in the analysis, i.e. a total of 222 soils samples from the six groups, the correlation coefficient (R^2^) increases to 0.92 with a similar RMSE (0.34). On the other hand, MAP has a statistical correlation with the abundance of Ia fraction, i.e. high abundance in Ia fraction corresponds to high MAP (Figs 4 and 5A). However, the correlation between CBT′ and MAP is relatively weak (R^2^ = 0.35).

The second Principal Component (PC 2) corresponds to 30% of total variations, and it aligns with MAT and MBT′_5ME_. As the plot (Fig. 5A) suggests, the abundance of IIa fraction is negatively correlated with air temperature (i.e. MAT and the MBT′_5ME_ index). The strength of the relationship between the brGDGTs derived MBT′_5ME_ and the observed MAT for the 104 soil samples from the humid and semi-humid regions is strong, as reflected in the high correlation coefficient (R^2^ = 0.82 for MAT and R^2^ = 0.84 for MAT_9months_) (Fig. 5D,E). However, the associated RMSE is large (RMSE = 3.26 °C for MAT and RMSE = 2.37 °C for MAT_9months_^19,27,39^. If the 222 soil samples from all six regions are included in the regression, the correlation coefficient between the brGDGTs derived MBT′_5ME_ and the observed MAT decreases significantly (R^2^ = 0.62) (Fig. 5F) with a slightly increase in RMSE (3.56 °C). In agreement with De Jonge^24^, the correlation coefficient (R^2^) (0.66) in the global data set is only marginally higher than those from regional calibrations. The significantly lower correlation coefficient for the combined data set in China implies that there is a certain level of uncertainty in the relationship between soil brGDGTs and air temperature. For palaeo-MAT reconstructions, therefore, choosing suitable calibration data set is important.

Nevertheless, the above analyses demonstrate that soil brGDGTs data with the separated 5-methyl and 6-methyl brGDGTs^24–26^ can be used for paleo-climate reconstructions because the fractional abundance of IIa′ and IIIa′ is useful in differentiating environmental conditions. In this study, the strong statistical relationship between the soil brGDGTs distributions and soil pH is confirmed. A weak relationship between the abundance of CBT′ fraction and MAP is revealed, and such relationship needs further investigations. Furthermore, While the CBT′ index is a suitable proxy for reconstructing soil pH, the MBT′_5ME_ index can be used as a proxy for MAT. Our regional calibrations provide not only strong correlations between MBT′_5ME_ and MAT, but also much reduced RMSEs in comparison to the global ones^17,24,28^. But as shown, caution should be applied when using soils from arid of semi-arid regions, due to the weaker correlation of brGDGTs with MAT in these environments.

## Methods

### Details of the sampling sites

The 47 new samples added in this study (Supplementary Table 1) were collected from humid subtropical climate condition. The west end of the sampling region (latitude 24°N and 30°N) is Guizhou Province in mid subtropical China, and the east is Zhejiang and Fujian Province (almost to the coast). At present, this climate zone is dominated by Asian monsoon, receiving 800~1600 mm precipitation annually. The January daily temperature varies between 0 °C and 15 °C. During summer months between May and September the daily high temperature often reaches 30 °C to 35 °C^33^. All samples from the 47 sites were obtained from local nature reserves to minimize the impacts of human activity. All the sampling sites are generally equal-distributed across the subtropical region.

### Soil pH measurements

Following Weijers^15^, triplicate portions of each sample were mixed with ultra-pure water at a ratio of 1:2.5 (g/ml); the pH value of the supernatant was measured using a pH meter (EZDO PH7200 waterproof pen) with a precision of ±0.01, and the mean (standard deviation, ±0.04) was recorded as the sample’s pH value.

### Environmental parameters

The climate information for each sampling site was obtained from the Worldclim dataset at a spatial resolution of 2~5 minutes. The software used for data extraction is DIVA-GIS. The MAT and MAP data are the average values for 1950~2000. The 9 months MAT data was obtained from mean air temperature ranging from March to November. All the collected data are calculated and adjusted according to sample altitude and the nearest meteorological station recorded data (http://cdc.cma.gov.cn/).

### Lipid extraction and GDGT analysis

Aliquots of the soil samples were prepared for GDGT analysis by freeze-dried at −18 °C in a refrigerator. The soils were ground into less than 200 mesh size. An organic solvent (9:1 dichloromethane: methanol) was added to each sample to extract organic compounds using ultrasonic extraction at least three times. *n*-Hexane was added to obtain the neutral extracts (at least three times). The neutral extracts were then purified and separated by silica-gel chromatography using hexane/DCM (9:1) and DCM/methanol (1:1) as subsequent eluents to separate into non-polar and polar fractions. The polar fraction containing the GDGTs was dried under nitrogen gas and then re-dissolved in hexane/isopropanol (99:1, v/v). The resulting samples were passed through a 0.45 µm polytetrafluoroethene filter before analysis.

The GDGT were analyzed at Tongji University by HPLC-atmospheric pressure chemical ionization-mass spectrometry (HPLC-APCI-MS), performed with a LC-MS. The 5-methyl and 6-methyl brGDGTs (Supplementary Fig. 1) were separated with an improved liquid chromatography method. Comparing with the new method with only one silica column, new method uses two coupled silica columns (each 150 mm × 2.1 mm, 1.9 μm; Thermo Finnigan; USA) at 40 °C using *n*-hexane and EtOAc (84:16 v/v) as elutes for pump A and pump B, respectively (modified from De Jonge^24^ and Yang^32^). Selected ion monitoring (SIM) was used to target specific [M + H]^+^, including those for the 15 brGDGTs ([M + H]^+^ 1050 IIIa III′a, 1048 IIIb III′b, 1046 IIIc III′c, 1036 IIa II′a, 1034 IIb II′b, 1032 IIc II′c, 1022 Ia, 1020 Ib and 1018 Ic). Whilst in old method, only 9 brGDGTs ([M + H]^+^ 1050 IIIa, 1048 IIIb, 1046 IIIc, 1036 IIa, 1034 IIb, 1032 IIc, 1022 Ia, 1020 Ib and 1018 Ic) can be detected.

The relative abundances of individual brGDGTs were calculated according to the integrated peak areas. MBT′_5ME_, CBT′ were calculated based on the specific brGDGT group (5- and 6-methyl brGDGTs), where MBT′_5ME_ = (Ia + Ib + Ic)/(Ia + Ib + Ic + IIa + IIb + IIc + IIIa) and CBT′ = ^10^log[(Ic + IIa′ + IIb′ + IIc′ + IIIa′ + IIIb′ + IIIc′)/(Ia + IIa + IIIa)]^18^. Replicate HPLC/APCI-MS analysis of samples showed the reproducibility of MBT′_5ME_ and CBT′ (Supplementary Table 1) brGDGTs to be ± 0.0019 and ± 0.018, respectively. For old method, the MBT′ and CBT calibrations are: MBT′ = (Ia + Ib + Ic)/(Ia + Ib + Ic + IIa + IIb + IIc + IIIa) and CBT = −^10^log[(Ib + IIb)/(Ia + IIa)]^22^.

The soil transfer functions of MBT′_5ME_-MAT and CBT′-pH are: MAT = −8.57 + 31.45 × MBT′_5ME_ (r^2^ = 0.66, RMSE = 4.8 °C) and pH = 7.15 + 1.59 × CBT′ (r^2^ = 0.85, RMSE = 0.52)^18^. The peat transfer functions of MBT′_5ME_-MAT_peat_ and CBT_peat_-pH are MAT_peat_ = 23.05 + 52.18 × MBT′_5ME_ (r^2^ = 0.76, RMSE = 4.7 °C) and pH = 8.07 + 2.49 × CBT_peat_ (r^2^ = 0.58, RMSE = 0.8)^17^. For the old method, the transfer functions are: MAT = 0.81–5.67 × CBT + 31.0 × MBT′ (r^2^ = 0.59, RMSE = 5.0 °C) and pH = 7.90–1.97 × CBT (r^2^ = 0.70, RMSE = 0.8)^22^.

### Statistical analyses

All statistical analyses (e.g factor analysis and linear regression analysis) were performed with the SPSS 19 software and ORINGIN PRO8 software. Significant differences between datasets are characterized by a Pearson’s coefficient (*p*-value) < 0.05.

## Supplementary information

supplementary figure 1

supplementary table 1

## Data Availability

The datasets generated during and/or analyzed during the current study are available in the Supplementary Table 1.
