# Supplementary material for: Distributions of soil branched glycerol dialkyl glycerol tetraethers from different climate regions of China
Source: Sci Rep. 2019 Feb 26;9:2761. doi: 10.1038/s41598-019-39147-9 (PMC6391447; doi:10.1038/s41598-019-39147-9)
Supplement: Supplementary file 1 — supplementary figure 1 [file 41598_2019_39147_MOESM1_ESM.pdf]

Distributions of soil branched glycerol dialkyl glycerol tetraethers from different climate regions of China

M. Wang[1,2], Z. Zheng[1], Y. Zong[2], M. Man[1], L. Tian[1]

[1] School of Earth Science and Geological Engineering, Sun Yat-Sen University, Guangzhou, China. [2] Department of Earth Sciences, The University of Hong Kong, Hong Kong SAR, China. Correspondence should be addressed to Y.Z. (email: yqzong@hku.hk)

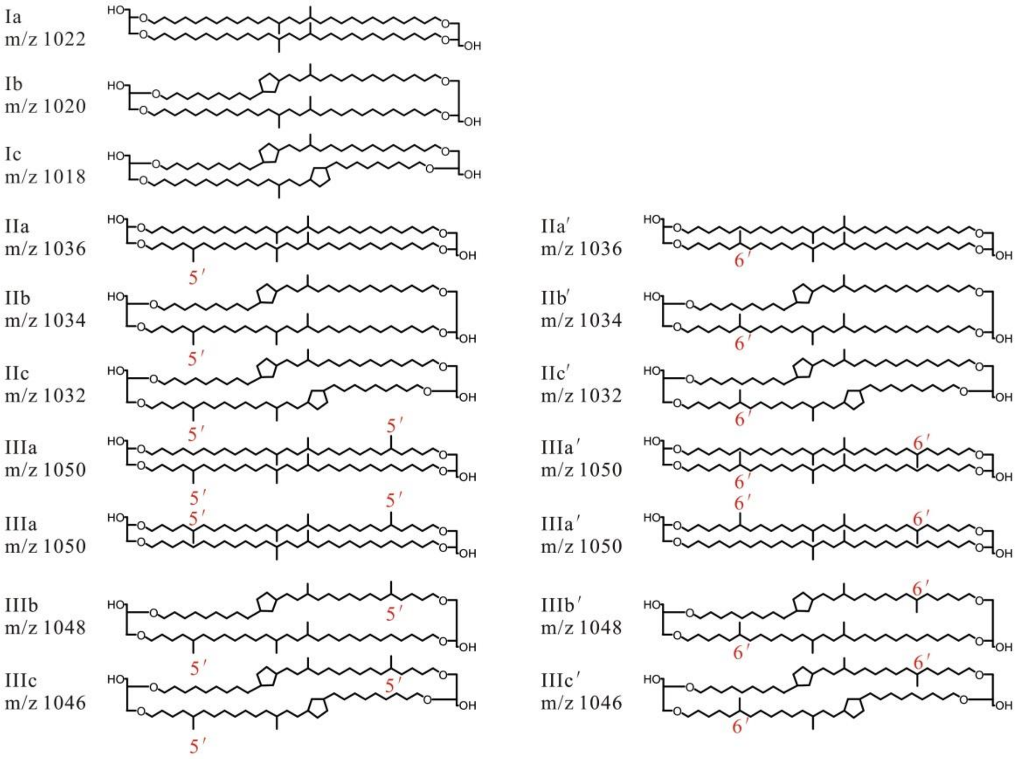

Supplementary Fig. 1 Chemical structures of branched GDGTs (I-III). Separated 5-methyl and 6-methyl brGDGTs based on the improved liquid chromatography method are shown in red.
