# Supplementary material for: Distributions of soil branched glycerol dialkyl glycerol tetraethers from different climate regions of China
Source: Sci Rep. 2019 Feb 26;9:2761. doi: 10.1038/s41598-019-39147-9 (PMC6391447; doi:10.1038/s41598-019-39147-9)
Supplement: Supplementary file 2 — supplementary table 1 [file 41598_2019_39147_MOESM2_ESM.pdf]

Distributions of soil branched glycerol dialkyl glycerol tetraethers from different climate regions of China

M. Wang[1,2], Z. Zheng[1], Y. Zong[2], M. Man[1], L. Tian[1]
1 School of Earth Science and Geological Engineering, Sun Yat-Sen University, Guangzhou, China. 2 Department of Earth Sciences, The University of Hong Kong, Hong Kong SAR, China. Correspondence should be addressed to Y.Z. (email: yqzong@hku.hk)

Environmental data

No.	Depth(cm)	LAT (N)	LONG (E)	pH	MAT/ ℃	9 months MAT/ ℃	MAP/mm
1	0-10	26.8	119.1	5.3	14.9	18.8	1843
2	0-10	28.4	119.3	5.6	14.2	18.0	1818
3	0-10	26.1	104.8	5.4	12.7	16.6	1230
4	0-10	27.7	119.6	5.2	12.6	16.7	1991
5	0-10	26.5	114.1	5.7	12.2	17.0	1827
6	0-10	26.3	106.2	5.0	15.9	18.8	1272
7	0-10	26.6	114.1	6.9	14.3	15.4	1818
8	0-10	29.5	120.5	5.2	13.1	17.8	1629
9	0-10	26.4	108.2	5.8	12.1	14.0	1399
10	0-10	28.4	114.1	5.7	11.0	18.7	1772
11	0-10	26.5	114.2	5.8	14.6	20.0	1648
12	0-10	26.1	110.4	5.2	10.5	16.8	1620
13	0-10	27.0	118.9	5.2	13.8	16.8	1993
14	0-10	24.5	112.6	6.9	18.3	20.7	1574
15	0-10	24.7	117.1	6.0	19.9	20.8	1689
16	0-10	26.6	116.1	6.6	18.5	21.8	1692
17	0-10	26.1	104.8	4.8	11.3	19.8	1234
18	0-10	26.1	104.8	6.7	11.3	19.8	1234
19	0-10	26.1	104.8	5.2	12.7	16.6	1230
20	0-10	27.2	116.9	6.3	18.7	22.1	1732
21	0-10	26.5	114.1	5.2	10.8	15.4	1928
22	Surface soil	27.9	108.7	5.4	14.3	15.8	1328
23	Surface soil	27.9	108.7	5.4	6.3	17.1	1662
24	Surface soil	27.9	108.7	5.1	12.9	16.0	1245
25	Surface soil	27.9	108.7	5.6	13.5	15.2	1212
26	Surface soil	27.9	108.7	5.1	13.1	18.7	2081
27	Surface soil	27.9	108.7	5.4	11.2	12.3	2233
28	Surface soil	27.9	108.7	5.0	12.7	18.7	1224
29	Surface soil	27.9	108.7	5.1	15.6	18.7	1234
30	Surface soil	27.9	108.7	5.4	12.4	20.8	1425
31	Surface soil	26.4	108.2	5.2	15.7	15.5	1576
32	Surface soil	29.5	120.5	5.2	12.6	18.0	1818
33	Surface soil	29.1	107.2	5.4	14.2	14.1	1827
34	Surface soil	26.0	104.7	5.4	15.2	15.6	1566
35	Surface soil	28.9	118.1	5.0	12.2	19.2	1845
36	Surface soil	27.8	117.8	5.5	13.2	17.0	1811
37	Surface soil	26.6	107.6	5.0	12.4	15.8	1778
38	Surface soil	26.1	104.8	5.4	15.1	19.9	1701
39	Surface soil	30.2	119.5	4.0	14.7	18.9	2597
40	Surface soil	27.8	110.8	4.4	15.3	18.2	2765
41	Surface soil	28.4	119.3	4.1	14.7	17.6	2898
42	Surface soil	26.5	114.0	4.8	14.1	17.0	2605
43	Surface soil	30.3	119.4	4.3	13.5	16.4	2495
44	Surface soil	28.3	119.3	4.4	12.9	15.8	2379
45	Surface soil	26.1	113.9	4.6	12.3	15.2	2267
46	Surface soil	26.5	114.1	5.4	11.7	14.6	2158

47	Surface soil	27.6	114.2	4.0	11.0	13.9	2028

Fractional abundances

No.	f (IIIa)	f (IIIa')	f (IIIb)	f (IIIb')	f (IIIc)	f (IIIc')	f (IIa)	f (IIa')
1	0.8	0.1	0.0	0.0	0.0	0.0	12.3	2.0
2	1.4	0.2	0.0	0.0	0.0	0.0	16.0	4.2
3	2.6	0.1	0.1	0.0	0.1	0.0	29.6	3.3
4	3.0	0.1	0.0	0.0	0.0	0.0	22.7	2.1
5	5.1	0.7	0.0	0.0	0.0	0.0	25.8	5.8
6	0.9	0.0	0.0	0.0	0.0	0.0	14.4	1.6
7	3.4	1.3	0.2	0.0	0.0	0.0	22.9	10.4
8	5.0	0.3	0.0	0.0	0.0	0.0	26.3	4.0
9	8.4	1.2	0.1	0.0	0.1	0.0	31.4	7.2
10	2.6	0.1	0.0	0.0	0.0	0.0	23.1	2.7
11	2.1	0.3	0.0	0.0	0.0	0.0	21.2	5.5
12	1.7	0.0	0.0	0.0	0.0	0.0	23.9	2.0
13	5.8	0.3	0.0	0.0	0.0	0.0	28.5	4.4
14	1.3	0.3	0.2	0.0	0.1	0.0	16.3	15.5
15	0.8	0.4	0.0	0.0	0.0	0.0	13.7	7.7
16	1.5	0.3	0.1	0.0	0.0	0.0	15.8	6.6
17	3.0	0.1	0.0	0.0	0.0	0.0	24.5	2.7
18	1.9	1.1	0.0	0.0	0.0	0.0	18.0	10.6
19	0.6	0.2	0.1	0.0	0.3	0.0	14.6	1.5
20	1.4	0.5	0.0	0.0	0.0	0.0	15.0	6.0
21	2.6	0.1	0.0	0.0	0.0	0.0	21.5	1.6
22	1.6	0.6	0.0	0.0	0.0	0.0	20.7	7.5
23	1.0	0.3	0.0	0.0	0.0	0.0	13.9	4.7
24	1.1	0.1	0.0	0.0	0.1	0.0	15.6	3.4
25	1.1	0.9	0.0	0.0	0.1	0.0	14.1	10.5
26	1.2	0.0	0.0	0.0	0.0	0.0	15.9	3.1
27	1.9	0.5	0.0	0.0	0.0	0.0	14.3	6.5
28	0.5	0.0	0.0	0.0	0.0	0.0	9.2	2.3
29	1.0	0.0	0.0	0.0	0.0	0.0	15.3	2.2
30	0.8	0.4	0.0	0.0	0.0	0.0	8.3	5.4
31	0.9	0.0	0.1	0.0	0.1	0.0	10.3	2.2
32	1.1	0.3	0.0	0.0	0.0	0.0	13.8	5.4
33	2.0	0.3	0.0	0.0	0.1	0.0	19.9	3.2
34	0.8	0.1	0.0	0.0	0.0	0.0	11.8	3.9
35	0.1	0.0	0.0	0.0	0.0	0.0	3.2	0.9
36	0.6	0.6	0.1	0.0	0.0	0.0	8.7	5.9
37	0.5	0.0	0.0	0.0	0.0	0.0	8.5	2.0
38	0.9	0.2	0.0	0.0	0.0	0.0	12.2	3.8
39	0.4	0.0	0.0	0.0	0.0	0.0	7.6	0.3
40	0.9	0.1	0.0	0.0	0.0	0.0	13.1	0.7
41	0.8	0.1	0.0	0.0	0.0	0.0	13.2	0.3
42	0.7	0.1	0.0	0.0	0.0	0.0	13.0	0.6
43	0.8	0.1	0.1	0.0	0.2	0.0	11.6	0.6
44	2.1	0.5	0.0	0.0	0.0	0.0	23.4	1.8
45	1.9	0.3	0.0	0.0	0.0	0.0	24.8	1.1
46	3.8	1.9	0.4	0.5	1.1	0.3	24.9	5.7
47	1.7	0.4	0.0	0.0	0.0	0.0	23.5	3.8

f (IIb)	f (IIb')	f (IIc)	f (IIc')	f (Ia)	f (Ib)	f (Ic)
0.3	0.1	0.2	0.0	80.0	3.0	1.4
0.7	0.4	0.3	0.0	69.1	6.1	1.5
1.5	0.6	1.9	0.0	52.2	2.8	5.3
0.7	0.3	0.4	0.0	66.5	3.2	1.2
1.4	0.9	0.6	0.0	54.4	4.1	1.2
0.5	0.2	0.5	0.0	76.4	2.5	3.0
4.0	2.2	0.5	0.1	42.9	10.3	1.7
1.0	0.5	0.6	0.0	58.0	3.1	1.0
1.3	0.4	0.4	0.0	45.7	3.0	0.8
0.7	0.2	0.3	0.0	64.5	4.7	1.0
1.7	0.7	0.3	0.0	58.1	8.4	1.6
0.6	0.3	0.6	0.0	58.4	3.7	8.7
0.6	0.4	0.1	0.0	57.3	2.3	0.2
2.3	2.2	0.2	0.0	50.4	9.9	1.4
0.8	0.3	0.2	0.0	68.6	6.5	1.0
2.4	0.9	0.3	0.0	57.9	12.4	1.7
0.4	0.2	0.2	0.0	67.1	1.5	0.3
1.2	1.8	0.2	0.2	55.1	8.5	1.4
0.6	0.3	0.8	0.0	75.1	3.7	2.3
1.5	0.7	0.4	0.0	64.5	8.0	1.9
0.8	0.1	0.3	0.0	66.4	4.7	2.0
0.4	0.8	0.1	0.0	63.6	3.9	0.9
0.2	0.3	0.0	0.0	74.7	4.1	0.9
0.1	0.1	0.0	0.0	78.5	0.9	0.2
0.3	0.7	0.1	0.0	69.8	2.0	0.4
0.1	0.1	0.1	0.0	78.0	0.8	0.5
0.1	0.3	0.1	0.0	74.3	1.8	0.1
0.1	0.1	0.0	0.0	85.8	1.4	0.4
0.3	0.1	0.2	0.0	76.7	3.2	1.0
0.9	1.7	0.2	0.1	73.0	6.7	2.4
0.3	0.0	0.5	0.0	81.7	2.1	1.8
0.9	0.8	0.3	0.0	67.2	8.7	1.6
0.5	0.3	0.2	0.0	68.0	4.6	0.8
0.1	0.0	0.0	0.0	82.2	0.8	0.3
0.0	0.0	0.0	0.0	94.6	0.4	0.7
0.5	0.7	0.1	0.0	74.7	7.7	0.5
0.0	0.1	0.0	0.0	88.0	0.6	0.3
0.4	0.6	0.1	0.0	73.7	6.0	2.1
0.1	0.0	0.0	0.0	90.2	0.9	0.5
0.1	0.1	0.0	0.0	82.7	1.7	0.4
0.2	0.0	0.1	0.0	83.2	1.6	0.6
0.2	0.0	0.1	0.0	83.0	1.6	0.8
0.1	0.0	0.0	0.0	85.4	0.8	0.2
0.5	0.3	0.2	0.0	67.7	3.0	0.6
0.6	0.1	0.3	0.0	67.1	2.8	0.9
1.4	1.5	0.8	0.1	49.4	5.8	2.4
0.4	0.2	0.1	0.0	69.0	0.8	0.2
